# Computational screening combined with well-tempered metadynamics simulations identifies potential TMPRSS2 inhibitors

**DOI:** 10.1038/s41598-024-65296-7

**Published:** 2024-07-13

**Authors:** C. S. Sharanya, D. Sasikala Wilbee, Shijulal Nelson Sathi, Kathiresan Natarajan

**Affiliations:** 1 Transdisciplinary Biology, Rajiv Gandhi Centre for Biotechnology, Thiruvananthapuram, Kerala India; 2 College of Pharmaceutical Sciences, Government Medical College, Thiruvananthapuram, Kerala India; 3 Bioinformatics Laboratory, Rajiv Gandhi Centre for Biotechnology, Thiruvananthapuram, Kerala India

**Keywords:** TMPRSS2, Virtual screening, Molecular docking, Molecular dynamics, e-pharmacophore, MM-GBSA, Well-tempered metadynamics, Computational biology and bioinformatics, Drug discovery

## Abstract

Type-II transmembrane serine proteases are effective pharmacological targets for host defence against viral entry and in certain cancer cell progressions. These serine proteases cleave viral spike proteins to expose the fusion peptide for cell entry, which is essential to the life cycle of the virus. TMPRSS2 inhibitors can also fight against respiratory viruses that employ them for cell entry. Our study combining virtual screening, all-atom molecular dynamics, and well-tempered metadynamics simulation identifies vicenin-2, neohesperidin, naringin, and rhoifolin as promising TMPRSS2 antagonists. The binding energies obtained are − 16.3, − 15.4, − 13.6, and − 13.8 kcal/mol for vicenin-2, neohesperidin, naringin, and rhoifolin respectively. The RMSD, RMSF, PCA, DCCM, and binding free energy profiles also correlate with the stable binding of these ligands at the active site of TMPRSS2. The study reveals that these molecules could be promising lead molecules for combating future outbreaks of coronavirus and other respiratory viruses.

## Introduction

The COVID-19 pandemic has led to significant loss of life globally^1,2^, making it challenging to provide preventive measures for vulnerable and high-risk populations, particularly individuals with weakened immune systems who may not mount an adequate response to vaccines^3,4^. Currently, monoclonal antibodies are the only available prophylactic option for these vulnerable and high-risk groups, but their effectiveness is hindered by the virus's ability to evolve and evade neutralization^4,5^. Mutations in the spike protein of SARS-CoV-2 have been found to evade the immune response and enhance transmission^6,7^.

Recent research has shown that three or four doses of mRNA vaccine did not generate strong neutralization against the BA.4/5 variant^8,9^ and highlights the need for bivalent vaccines that target both the original and the BA.4/5 spike proteins^10^. The emergence of new sub-lineages like Omicron, which have additional spike mutations warrants new vaccine research^11^. As there are no other approved drugs for pharmacological prevention, there is an urgent need for novel drug candidates that can reduce the severity of the disease, exhibit lower susceptibility to viral resistance, and be suitable for implementation in the healthcare systems of low and middle-income countries.

TMPRSS2, or transmembrane protease serine 2 plays a crucial role in SARS-CoV-2 viral entry into human cells. It facilitates viral entry by cleaving its spike protein after binding to the ACE2 receptor^12–14^. This makes it an attractive target for COVID-19 treatments, as inhibiting TMPRSS2 function could prevent or reduce viral infection. TMPRSS2 has also been implicated in other diseases, including prostate cancer and influenza viral infections^15–20^. TMPRSS2 functions by proteolytically cleaving fusion glycoproteins of the Sendai virus (SeV), human metapneumovirus (HMPV), and human parainfluenza 1, 2, 3, 4a, and 4b viruses (HPIV), as well as the spike glycoproteins of the human coronaviruses-EMC (HCoV-EMC) and 229E (HCoV-229E)^21,22^. It also plays a key role in the pathogenesis and dissemination of influenza A viruses (strains H1N1, H3N2, and H7N9) by cleaving and activating the viral hemagglutinin (HA) protein^23–25^.

The human TMPRSS2 gene belongs to the transmembrane serine protease family, which encodes a protein consisting of 492 amino acids. It consists of a signal peptide, an extracellular adhesion domain, a transmembrane domain, and a serine protease domain. The extracellular adhesion domain comprises a unique scavenger receptor cysteine-rich (SRCR) domain^15,26,27^ while the serine protease domain contains a catalytic triad of residues (Ser441-His296-Asp345) essential for its protease activity. TMPRSS2 can activate proteins involved in the differentiation of epithelial cells in the prostate and lung, where it cleaves and activates several transcription factors and growth factors. It can regulate the activity of voltage-gated sodium channels and ionotropic glutamate receptors by cleaving their extracellular domains. TMPRSS2 also plays a role in the fibrinolytic system by activating plasminogen to plasmin to dissolve blood clots. Thus, TMPRSS2 has diverse functions in the human body and is crucial for various physiological and pathological processes^13,28–30^.

Structure-based drug design using virtual screening, molecular docking, molecular dynamics and well-tempered metadynamics simulation has proven to help identify novel lead molecules for the possible treatment of several diseases. Earlier studies by Haridas et al., 2021^31^showed that *Citrus medica* and *Zingiber officinale* contain a range of bioactive compounds with potential therapeutic benefits and are useful in herbal formulations used for managing contagious fever, and other symptoms similar to COVID-19. In a similar study, high-throughput virtual screening was used to identify lead molecules against the SARS-CoV-2 spike protein. Acetyl-11-keto-boswellic acid (AKBA) derivatives have shown promising results both in silico and *in vitro*^32^. Computer-aided drug design approaches such as Molecular Docking, Molecular Dynamics, MM-PBSA, Pharmacokinetics, and Density Functional Theory (DFT) have also been used for the screening of several pyridine derivatives against viral proteases. The binding stability of the pyridine derivatives and the protease enzyme was studied using MD simulations^33^. Moreover, two novel andrographolide derivatives have been identified as drug leads against Nsp14 and Nsp16 through MD simulations which has shown that the binding energies are lower than the control^34^.

In this work, we performed structural modeling of the ectodomain of serine protease TMPRSS2, followed by a structure-based virtual screening combining all-atom Molecular Dynamics (MD) and well-tempered metadynamics simulations to evaluate the binding efficacy of lead molecules. This study identifies natural products such as vicenin-2, neohesperidin, naringin and rhoifolin as promising TMPRSS2 antagonists which can act as SARS-CoV-2 viral entry inhibitors.

## Results and discussion

**Figure 1:**
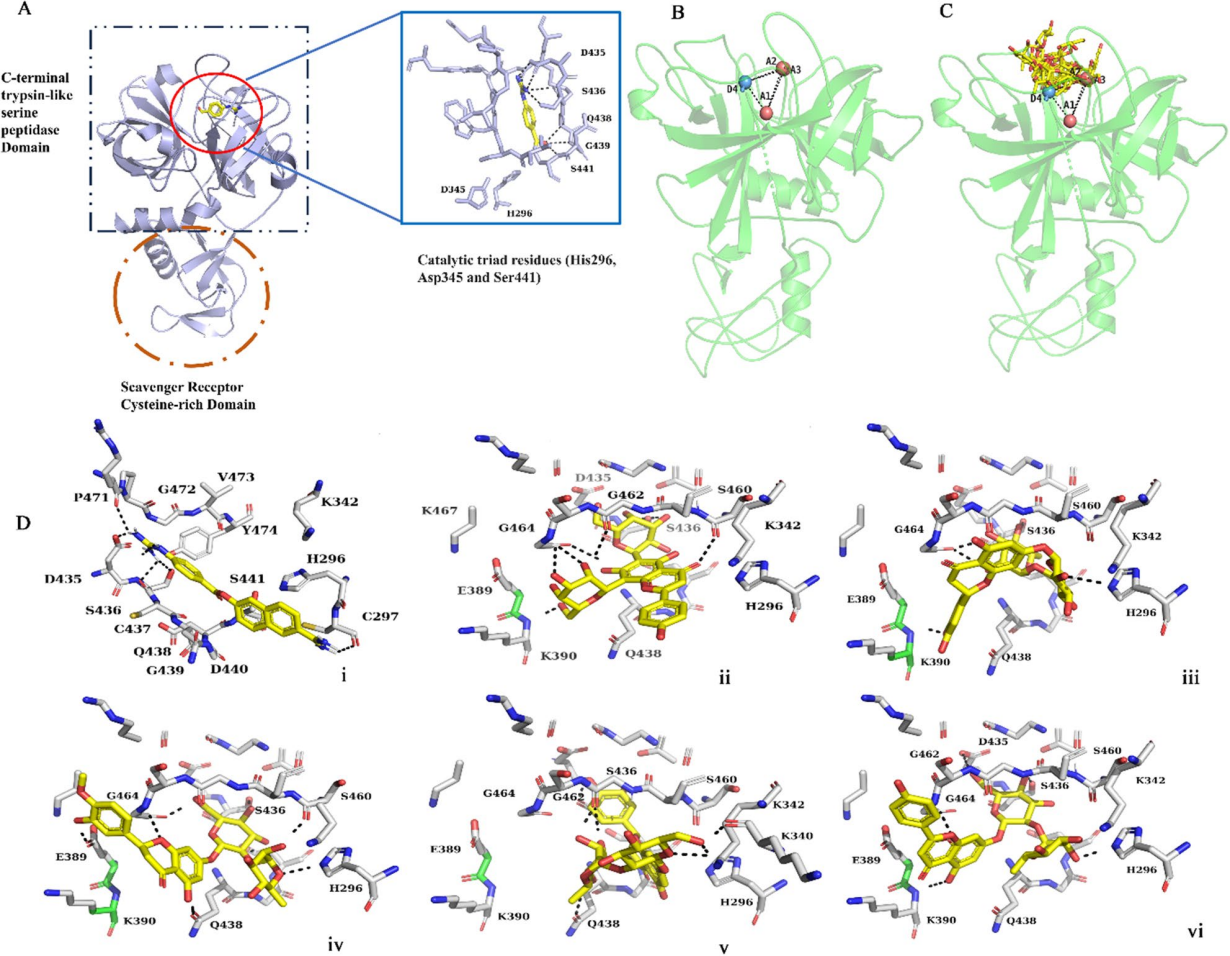
(**A**) Structure of TMPRSS2 ectodomain complex with nafamostat. The inset shows nafamostat interactions and the catalytic triad of TMPRSS2 (PDB ID: 7MEQ), (**B**) The schematic of the e-pharmacophore hypothesis (A1A2A3D4) and the view of the protein active site, (**C**) pharmacophore-based screening of ligand molecules, (**D**) ligand interactions after XP docking; (i) TMPRSS2-nafamostat complex; (ii) TMPRSS2-vicenin-2 complex; (iii) TMPRSS2-hesperidin complex; (iv) TMPRSS2-neohesperidin complex; (v) TMPRSS2-naringin complex; and (vi) TMPRSS2-rhoifolin complex.

The serine protease domain of TMPRSS2 is highly conserved and confirms the canonical chymotrypsin/trypsin fold harboring the catalytic Ser-His-Asp triad^26^ (Fig. 1A). Our study identifies the active site blockers of TMPRSS2 through virtual screening and validates their interaction strategies, and binding free energies through all-atom MD and well-tempered metadynamics simulations.

### Homology modelling

The human TMPRSS2 ectodomain structure was homology-modeled, showing 98% sequence identity with the template PDB ID: 7MEQ. The Ramachandran plot revealed that 95% of amino acids are in the allowed region. A QMEAN global score of 0.9 ± 0.05 and a MolProbity score of 1.24 also ensured the quality of the model (Supplementary Figure 1). The Protein Reliability Report in Maestro was also used to assess the reliability of the model (Supplementary Figure 1). The model showed *in-silico* parametric stability suitable for further *in-silico* analysis.

### Generation of e-pharmacophore model

We used the homology-modeled ectodomain structure of TMPRSS2 to generate an energy-optimized pharmacophore hypothesis (e-pharmacophore). The protein structure was pre-processed using the protein preparation wizard^35^ of the Schrodinger suite and subjected to restrained energy minimization using the OPLS4^36^ force field. The e-pharmacophore model was developed using the ‘Develop Pharmacophore from protein–ligand complex’ option in the Phase module of the Schrodinger suite. The amino acids in the receptor binding site were analyzed, and a four-featured pharmacophore hypothesis, AAAD, was generated (Fig. 1B, C) and used as a 3-D search query to screen the molecules from the natural product database LOTUS^37^, ChEMBL (https://www.ebi.ac.uk/chembl/) database, and OTAVA chemicals (https://www.otavachemicals.com/) with similar pharmacophore features. The phase module analyzed the fitness of the compounds with the query hypothesis and ranked them based on the fitness scores, selecting compounds for virtual screening.

All molecular docking experiments were carried out using the GLIDE^38,39^ module of Maestro 11.4 software. The catalytic centre amino acids of TMPRSS2 (Ser441-His296-Asp345) were centered to create a receptor grid with a grid box dimension of 15 × 15 × 15 Å. The ligand molecules were ranked based on their binding affinities using GLIDE scores (G-scores). High-throughput virtual screening (HTVS) was used for the initial screening, followed by standard precision (SP) docking and additional extra precision (XP) docking. Five molecules (vicenin-2, neohesperidin, naringin, rhoifolin, and hesperidin) selected from the XP docking had a more significant docking score than the well-known inhibitor of TMPRSS2, nafamostat (Table 1).

**Table 1:** Molecules with the highest binding energy after extra-precision (XP) docking.

Ligand molecules	Structure	Fitness score	Glidescore (kcal/mol)	Binding energy (kcal/mol)
Nafamostat mesylate		1.214	− 6.815	− 55.49
Neohesperidin		1.294	− 9.748	− 69.36
Vicenin-2		1.416	− 10.314	− 59.79
Naringin		1.181	− 9.845	− 50.84
Hesperidin		1.250	− 9.918	− 43.22
Rhoifolin		1.328	− 8.793	− 37.68

The results showed that vicenin-2, a flavonoid glycoside, has a higher glide score (− 10.314 kcal/mol) than nafamostat (− 6.815 kcal/mol). Nafamostat is a synthetic serine protease inhibitor that has been proven to be effective against systemic inflammation, certain types of cancer and pancreatitis. It has also been recently reported to be active against SARS CoV-2 by inhibiting the TMPRSS2^40^. However, its easy hydrolysability and poor specificity reduce its efficacy. Here, we used nafamostat as a positive control. The docking studies showed that nafamostat interacts with TMPRSS2 via Ser436, Cys437, Pro471 and Cys297 through hydrogen bonding, and a salt bridge is formed between Asp435 and the guanidino group of nafamostat. 6-amidino-2-naphthol of nafamostat forms the pi-cationic and pi-pi stacking interaction with His296 (Fig. 1D i). Vicenin-2 forms hydrogen bond interactions with TMPRSS2 residues Lys390, Ser436, Cys437, and Gly462 through the glucopyranosyl moiety (Fig. 1D ii). Hesperidin forms hydrogen bond interactions with Ser436 and Gly464 (Fig. 1D iii). Neohesperidin forms hydrogen bond interactions with Ser460, His296, Gly464 and Glu389 (Fig. 1D iv). Naringin makes hydrogen bonding interactions with Gly462, Gly464, Gln438 and Lys340, and hydrophobic contacts with Cys437, Trp461 and Cys465. It also forms a pi-pi stacking interaction with His296 (Fig. 1D v). Rhoifolin forms hydrogen bonds with Lys390, His296 and Gly464 (Fig. 1D vi). The results show that all these compounds interact with the active site residues through hydrogen bonding, hindering TMPRSS2 activity. The binding free energy of these high-scored molecules was calculated through the MM-GBSA (Molecular mechanics with generalised Born and surface area solvation) method. Nafamostat showed binding energy of − 55.49 kcal/mol, while the docked ligand complexes showed binding energy values of − 69.36 kcal/mol (neohesperidin), − 50.84 kcal/mol (naringin), − 59.79 kcal/mol (vicenin-2), − 43.22 kcal/mol (hesperidin), and − 37.68 kcal/mol (rhoifolin).

### MD simulations

**Figure 2:**
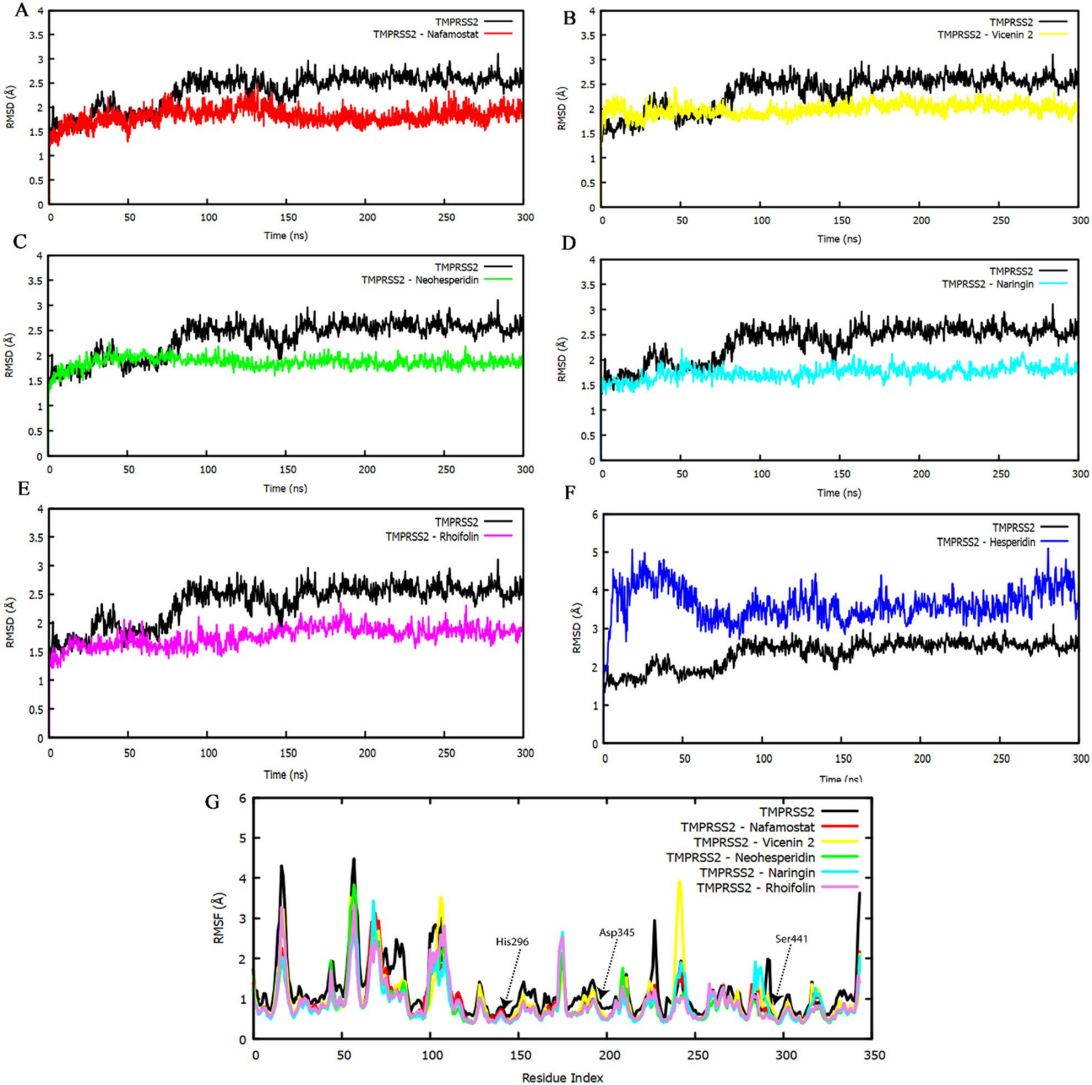
(**A–F**) Plot of RMSD distribution during 300 ns MD simulations, (**G**) The dynamic stability of the protein–ligand complex accessed by RMSF.

MD simulation is an important tool for studying the dynamic behavior of proteins. In this study, 300 ns MD simulations were conducted to investigate the stability of the TMPRSS2-ligand complexes that are identified through virtual screening. The binding stability of the ligand molecules (vicenin-2, hesperidin, naringin, neohesperidin, and rhoifolin) with TMPRSS2 was assessed through MD simulations. The conformational changes of the protein due to ligand binding can be expressed in terms of the root mean square deviation (RMSD). Comparison of the trajectories of apo-TMPRSS2 (without ligand) and ligand-bound TMPRSS2 simulations showed that apo-TMPRSS2 undergoes higher RMSD changes than the ligand-bound TMPRSS2, suggesting that these ligands are tightly binding to the TMPRSS2 active site restricting the motions of structural elements in the ectodomain. The RMSD changes for the C_α_ atoms were computed over a 300 ns MD simulation. The TMPRSS2-nafamostat, vicenin-2, neohesperidin, naringin, and rhoifolin complexes showed a mean RMSD profile below 2 Å (Fig. 2A–E), except for the TMPRSS2-hesperidin complex, which showed an RMSD profile above 4 Å (Fig. 2F), indicating that except for hesperidin, other molecules steadily bind to the ectodomain of TMPRSS2. It was observed that the apo-TMPRSS2 RMSD is below 3 Å, whereas other ligand-bound TMPRSS2 complexes have an RMSD below 2 Å. Apoprotein fluctuates up to 90 ns and then stabilizes after 100 ns at around 2.8 Å till the end of the simulation. In the ligand-bound TMPRSS2 complexes, except for the hesperidin-TMPRSS2 complex, the rest of the complexes didn’t show any significant deviations or fluctuations in RMSD, and the systems are fully stabilized up to 300 ns. It indicates that, except hesperidin, all other ligands make stable contacts with the active site of the TMPRSS2 ectodomain. Hence, the TMPRSS2-hesperidin complex is not considered for further analysis.

We analyzed the dynamics of the TMPRSS2 ectodomain using Root Mean Square Fluctuation (RMSF) analysis of the residues in TMPRSS2 ligand-bound complexes and apo-TMPRSS2. The stability of protein–ligand complexes is largely determined by individual interacting amino acids and their flexibility can be calculated through RMSF analysis. The magnitude of the RMSF value implies either high or low flexibility. High RMSF was found in flexible protein regions, with the most fluctuating parts being the loop regions. The overall values of RMSF fall within 4.5 Å. It was observed that the catalytic residues Ser441, His296, and Asp345, as well as the residues Asp435, Ser460, and Gly462, were less fluctuating due to strong protein–ligand contacts, indicating the structural stability of TMPRSS2-ligand complexes. A low RMSF value of these active site residues indicates stable interactions of these amino acids with the ligands during MD simulations. It was also noted that the N-terminal region of the TMPRSS2 ectodomain had higher residual fluctuations than the C-terminal region (Fig. 2G). The RMSD and RMSF analyses suggest that the TMPRSS2-ligand complexes (vicenin-2, neohesperidin, naringin, and rhoifolin) remained stable throughout the 300 ns MD simulations.

**Figure 3:**
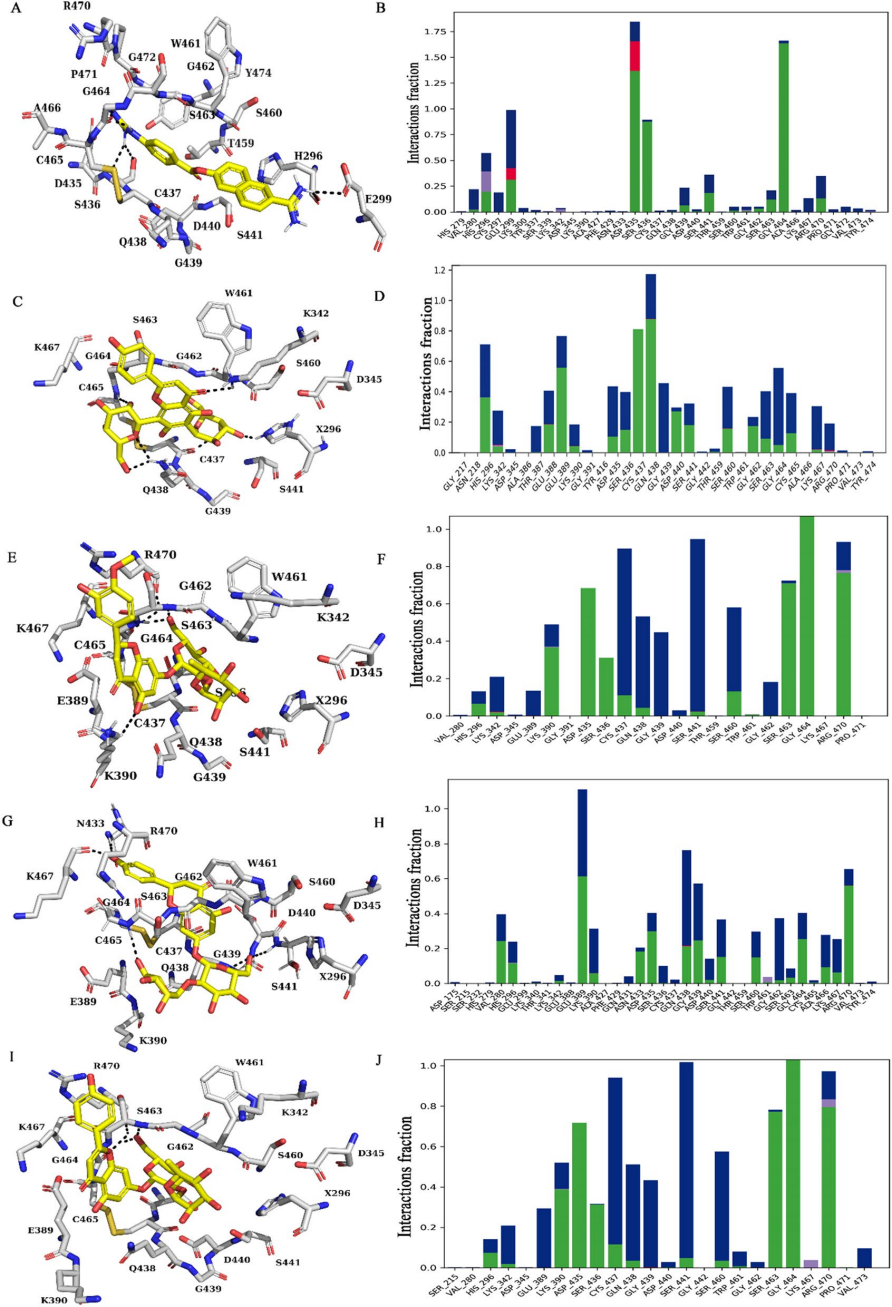
Ligand interactions and the bar plots showing TMPRSS2-ligands contacts averaged over 300 ns simulation time (**A–J**). (**A, B**) TMPRSS2-nafamostat complex, (**C, D**) TMPRSS2-vicenin-2 complex, (**E, F**) TMPRSS2-neohesperidin complex, (**G, H**) TMPRSS2-naringin complex and (**I, J**) TMPRSS2-rhoifolin complex. Ligands are represented as yellow sticks, and the interacting residues are represented as white sticks. The green color in the bar diagram represents hydrogen bonds, and the blue color represents water bridges.

We also conducted an interaction analysis on TMPRSS2-ligand complexes and found that both hydrogen bonding and hydrophobic interactions are stable. The intermolecular hydrogen bonds played a crucial role in determining the strength of the ligands binding to the protein (Fig. 3). Along the simulation trajectory, the guanidino group of nafamostat forms hydrogen bonds with the amino acid residues His296, Glu299, Asp435, Ser436, and Gly464. It also forms a salt bridge interaction with Asp435 throughout the simulation (Fig. 3A, B). The interactions of vicenin-2 with catalytic residues of TMPRSS2 during the MD simulations were found to be persistent. The hydrogen bonds stabilizing the binding of vicenin-2 and the residues His296, Glu389, Asp435, Ser436, Cys437, Gln438, Asp440, and Ser460 were persistent during the entire course of the MD simulations (Fig. 3C, D). The hydrogen bond interactions with the catalytic triad remain stable at most instants of MD simulations indicating that vicenin-2 might potentially interfere with the TMPRSS2 activity. Naringin also forms stabilizing hydrogen bonds with the catalytic residues Glu389, Glu439, Ser441, Gly464, and Lys467 (Fig. 3E, F). The neohesperidin-TMPRSS2 interaction was stabilized by hydrogen bond interactions with Lys390, Gly462, Gly464, and Arg470 (Fig. 3G, H). Similarly, the rhoifolin-TMPRSS2 interaction was also stabilized by hydrogen bond interactions with His296, Asp435, Ser436, Ser463, Gly464 and Arg470 (Fig. 3I, J). The interactions fraction indicates the fraction for which a particular amino acid is forming an interaction with the ligand during the simulation. The stacked bar charts are normalized throughout the trajectory and a value of 0.7 implies that 70% of the simulation time the specific interaction is maintained. Values over 1.0 are possible as some protein residue may make multiple contacts of the same subtype with the ligand. In Fig. 3B, Ser436, Gly464; in Fig. 3D, Cys437, Gln438; in Fig. 3F, Ser463, Gly464, Arg470; in Fig. 3H, Glu389, Arg470 and in Fig. 3J, Asp435, Ser463, Gly464, Arg470 implies that the specific interaction is maintained throughout 70% (0.7 on the graph) of the simulation time. The highest value for any given interaction is 1. If the interaction fraction is more than 1, the amino acid is likely forming more than one interaction with the ligand. In such a case, the interaction fraction can go up to 2 or 3 depending on the number of interactions it is forming. In Fig. 3B interaction fraction between Asp435 and Cys464 are more than 1, which means Asp435 and Cys464 have multiple interactions.

### Principal component analysis

Principal component analysis (PCA) is a widely used method to study MD simulation trajectories. PCA is used to determine the meaningful global motions in the protein during simulations. Protein dynamics involve changes in molecular structure and conformation over time, which are best represented by a vector space that spans a large number of dimensions equal to the number of degrees of freedom (DOF). PCA extracts the most important elements in the trajectory using a covariance matrix or a correlation matrix constructed from atomic coordinates that describe the accessible DOF of the protein^41–43^.

**Figure 4:**
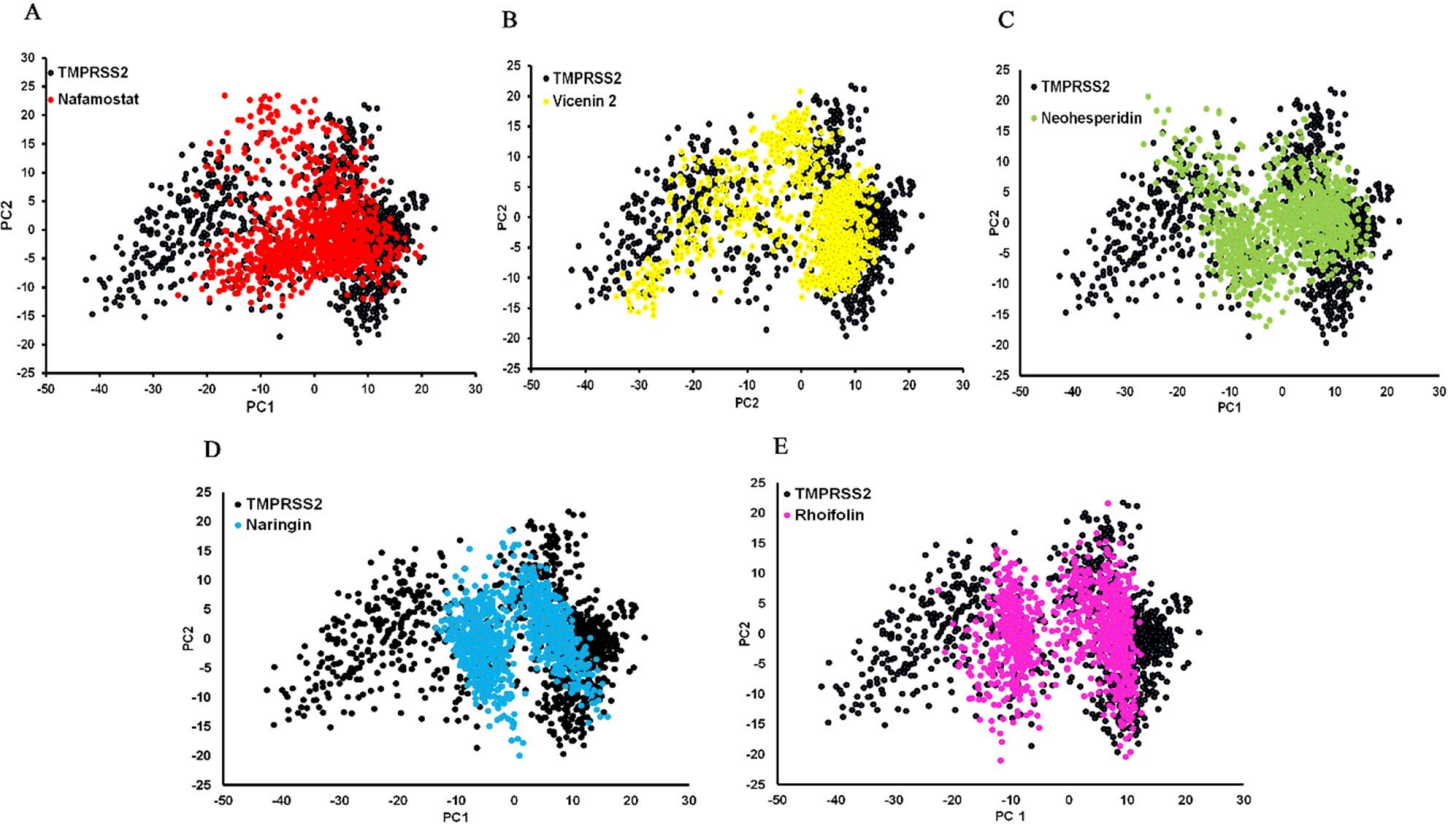
Projection of the TMPRSS2 ectodomain motion along PC1 and PC2 for TMPRSS2-Ligand complexes. (**A**) TMPRSS2-nafamostat complex, (**B**) TMPRSS2-vicenin 2 complex, (**C**) TMPRSS2-noehesperidin complex, (**D**) TMPRSS2-naringin complex, (**E**) TMPRSS2-rhoifolin complex.

To analyze the crucial collective motions of TMPRSS2 with and without ligands, covariance matrices of the C_α_ atoms were built to calculate principal modes. The positive values indicated correlated movements, while the negative values indicated anti-correlated motions between C_α_ atoms. Principal component 1 (PC1) represents the highest variation in the protein internal motion, and principal component 2 (PC2) accounts for the second most variation in the protein dynamics data. The TMPRSS2-nafamostat occupied most of the apo-TMPRSS2 conformational spaces (Fig. 4A) whereas the TMPRSS2-vicenin-2 complex (Fig. 4B) was completely superimposed in apo-TMPRSS2 conformational spaces, causing no structural changes. This represents the concerted motions of different regions of TMPRSS2 protein both in the nafamostat and vicenin 2 protein complexes. However, the other three lead compounds occupied fewer regions, indicating conformational changes during the binding process (Fig. 4C–E).

**Figure 5:**
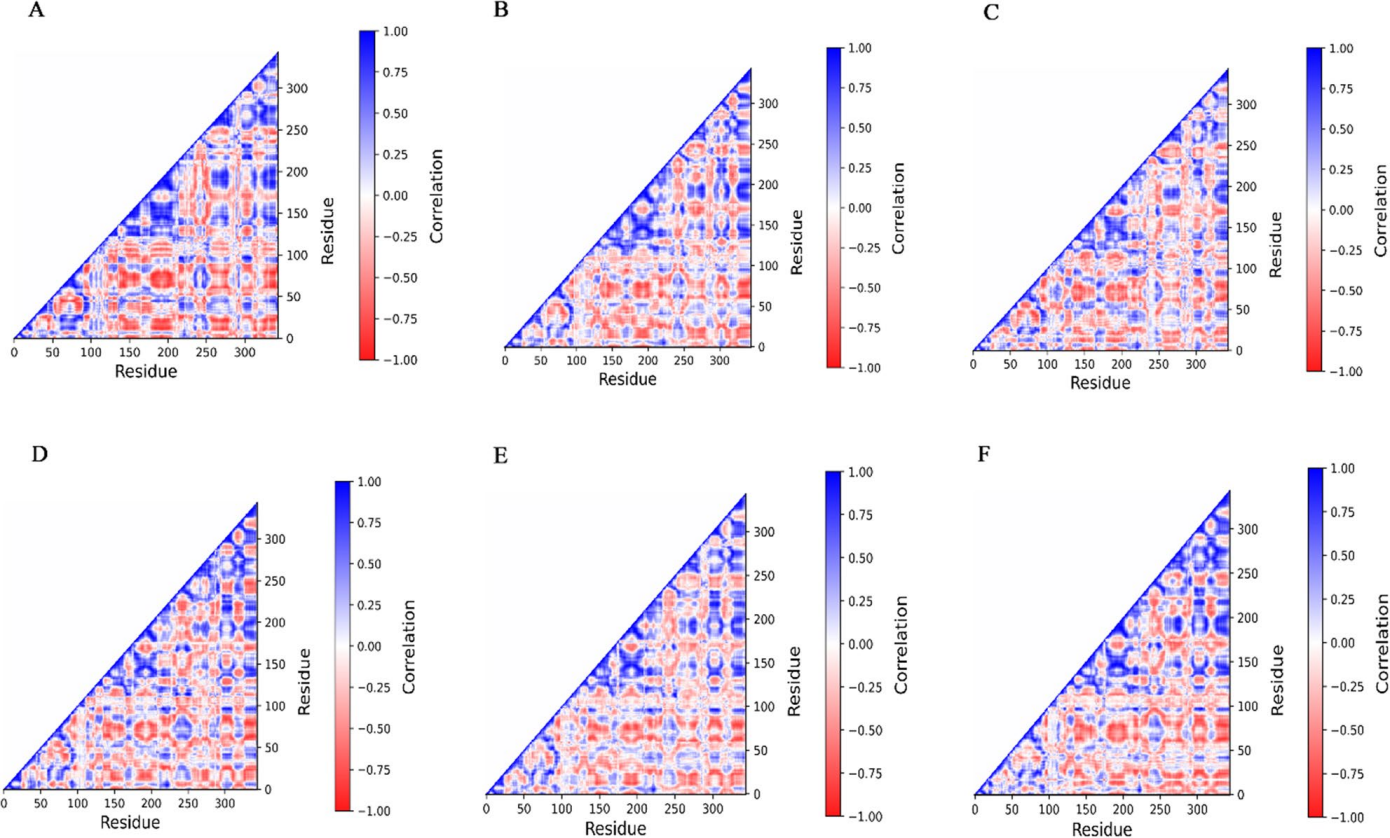
DCCM maps of Apo-TMPRSS2 and ligand-bound TMPRSS2 complexes. (**A**) Apo-TMPRSS2, (**B**) TMPRSS2 with nafamostat, (**C**) TMPRSS2 with vicenin-2, (**D**) TMPRSS2 with neohesperidin, (**E**) TMPRSS2 with naringin, and (**F**) TMPRSS2 with rhoifolin.The blue and red coloured patches represent strongly correlated (positive) and anticorrelated (negative) motions between residues, respectively.

Understanding the atomic motions and their collective behavior in proteins is crucial for understanding their biological function. This information can be extracted from MD trajectories. To determine the presence of correlated motions between the apo-TMPRSS2 and ligand-bound TMPRSS2 complexes, dynamic cross-correlation maps (DCCM) analysis was carried out on the C_α_ atoms in each simulation. DCCM maps illustrate the inter-residual movements calculated using MD trajectories. The blue and red coloured patches represent strongly correlated (positive) and anticorrelated (negative) motions between residues, respectively. Positive correlation demonstrates the strong stability of the protein–ligand binding (Fig. 5A–F). Positively correlated movements were particularly increased in the TMPRSS2-ligand bound complexes in the range of residues 150 to 350 (active site region), and a higher percentage of pairwise-connected residues indicated stable binding of ligands with the TMPRSS2.

### Binding free energy analysis

The binding free energy of the ligands was analyzed through the MM-GBSA method using MD trajectories extracted at intervals of 10 ns. The binding energy, along with other binding energy components such as electrostatic, covalent, Van der Waals, hydrogen bonding, lipophilic, Generalized Born electrostatic solvation, and pi-pi packing energies were calculated (Table 2). The highest binding energy was obtained for neo-hesperidin during the MD simulation (− 73.19 kcal/mol), followed by naringin (− 70.29 kcal/mol) and rhoifolin (− 71.50 kcal/mol). Vicenin-2 had a binding free energy of − 59.95 kcal/mol and the known inhibitor nafamostat had a binding free energy of − 58.39 kcal/mol. The results indicated that these molecules show higher binding free energy and stable interactions with the active site residues of TMPRSS2, indicating that these molecules could be potent inhibitors of TMPRSS2.

**Table 2:** Binding energy obtained post-MD simulation and the different energy components that contributed to the total binding energy.

Compounds	Binding energy (kcal/mol)	Coulomb	ΔG_Bind_Covalent	vdW	H-bond	Lipo	Solv GB	Packing
Nafamostat mesylate	− 58.39 ± 4.28	− 84.14 ± 26.35	4.00 ± 1.28	− 44.71 ± 2.87	− 3.98 ± 0.84	− 12.04 ± 0.93	85.11 ± 25.19	− 2.61 ± 0.96
Neo hesperidin	− 73.19 ± 5.61	− 36.37 ± 5.98	5.67 ± 2.08	− 55.49 ± 3.42	− 2.97 ± 0.64	− 12.64 ± 1.08	29.47 ± 4.28	− 0.77 ± 0.46
Rhoifolin	− 71.50 ± 5.16	− 35.50 ± 5.69	6.90 ± 1.41	− 55.47 ± 2.69	− 3.02 ± 0.55	− 11.87 ± 0.62	28.28 ± 2.70	− 0.81 ± 0.49
Naringin	− 70.29 ± 8.43	− 34.99 ± 8.17	3.47 ± 2.24	− 54.73 ± 3.64	− 2.93 ± 0.67	− 13.38 ± 0.89	32.42 ± 3.36	− 0.15 ± 0.42
Vicenin− 2	− 59.95 ± 7.07	− 34.56 ± 7.53	4.22 ± 1.64	− 51.26 ± 4.71	− 3.68 ± 1.07	− 10.30 ± 1.03	36.84 ± 4.15	− 1.19 ± 0.87

*Coulomb = Electrostatic, dG_Bind_Covalent = Covalent, vdW = Van der Waals, Hbond = Hydrogen bonding, Lipo = Lipophilic, Solv_GB = Generalized Born electrostatic solvation, Packing = pi-pi packing.

### TMPRSS2-spike interaction studies

We also created the TMPRSS2-Spike protein complex using the ClusPro 2.0 protein–protein docking webserver and docked these ligands to the catalytic site of TMPRSS2. For ClusPro protein–protein docking, the atomic coordinates of spike protein (PDB ID: 6CS2)^44^ were downloaded, and missing residues were modeled using UCSF Chimera^45^. The protein–protein docking was carried out using the modeled TMPRSS2 ectodomain (Supplementary Figure 2). The docked complex was selected for further docking with vicenin-2, neohesperidin, naringin, and rhoifolin using the GLIDE module of the Schrodinger suite. The glide docking scores for the molecules were − 13.294 kcal/mol, − 9.71 kcal/mol, − 9.337 kcal/mol and − 8.531 kcal/mol for vicenin-2, neohesperidin, naringin, and rhoifolin, respectively. Each of these molecules showed high binding affinity, suggesting that these molecules could inhibit the activity of TMPRSS2, which can be further validated by in vitro assays.

### Well-tempered metadynamics simulations

**Figure 6:**
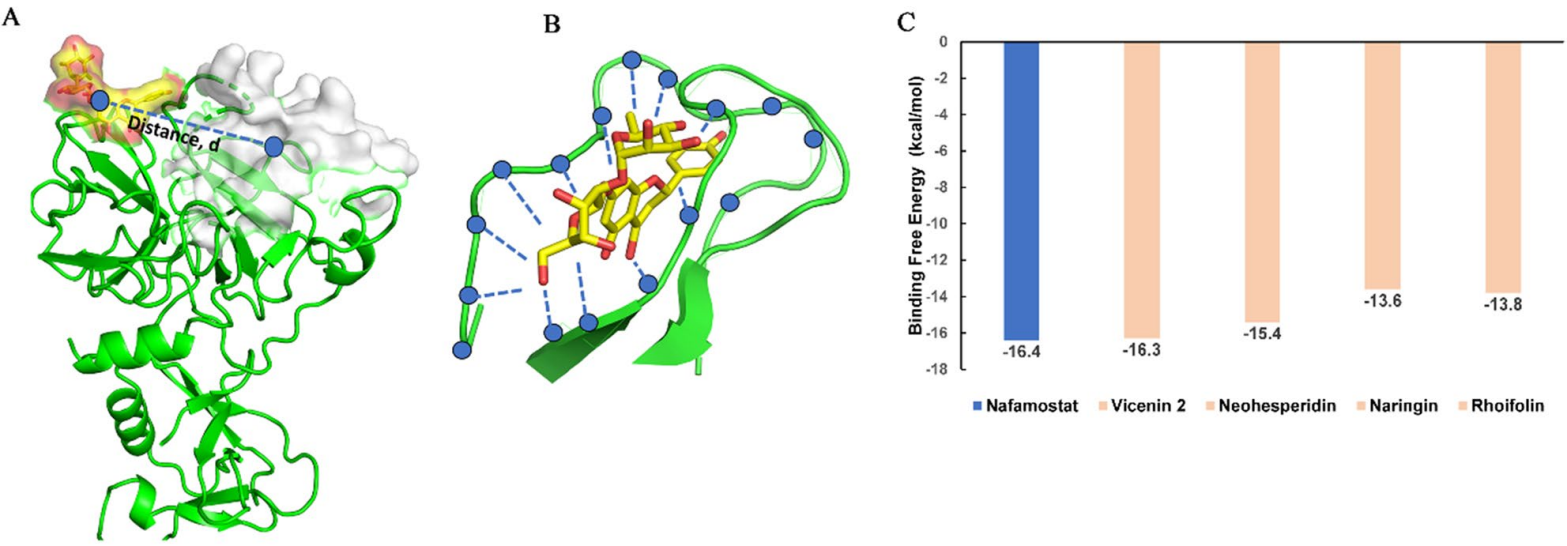
Schematic representation of the collective variables used for the well-tempered metadynamics simulations. (**A**) Collective variable, Distance (d) represents the distance between the centre of mass (COM) of the amino acids in the active site (white-coloured surface representation) and the COM of the drug (yellow-coloured surface representation), (**B**) the collective variable, number of contacts (Nc) represents the number of contacts (blue dots) between the drug and interacting atoms of the amino acids within a 4 Å radius of the atoms of the drug, (**C**) Bar diagram showing the binding free energy values of different small molecules (light orange) in comparison to that for the positive inhibitor Nafamostat mesylate (blue).

To understand the binding free energies of each of the ligands in the active site of the TMPRSS2, the ligand dissociation processes were carried out using well-tempered metadynamics simulations. The TMPRSS2-ligand complexes obtained after 300 ns MD simulations were subjected to well-tempered metadynamics simulations both in the case of nafamostat and the lead molecules vicenin-2, neohesperidin, naringin and rhoifolin using two collective variables: Distance, d and Number of Contacts, Nc. The schematic representation of collective variables is shown in Fig. 6A, B. The lower distance and higher number of contacts indicate the ligand's proximity to the protein, while the higher distance and lower number of contacts represent the farness of the ligand from the TMPRSS2 active site.

We compared the binding free energy of these ligands with respect to the positive control nafamostat (Fig. 6C). The results showed that the lead molecules exhibited a comparable binding affinity towards TMPRSS2. The binding free energy of vicenin-2 (-16.3 kcal/mol) is nearly identical to that of nafamostat. All other lead molecules also showed comparable binding free energy, like nafamostat. The one-dimensional (1D) free energy plot for all lead molecules compared to the TMPRSS2 inhibitor nafamostat is shown in Supplementary Figure 3.

**Figure 7:**
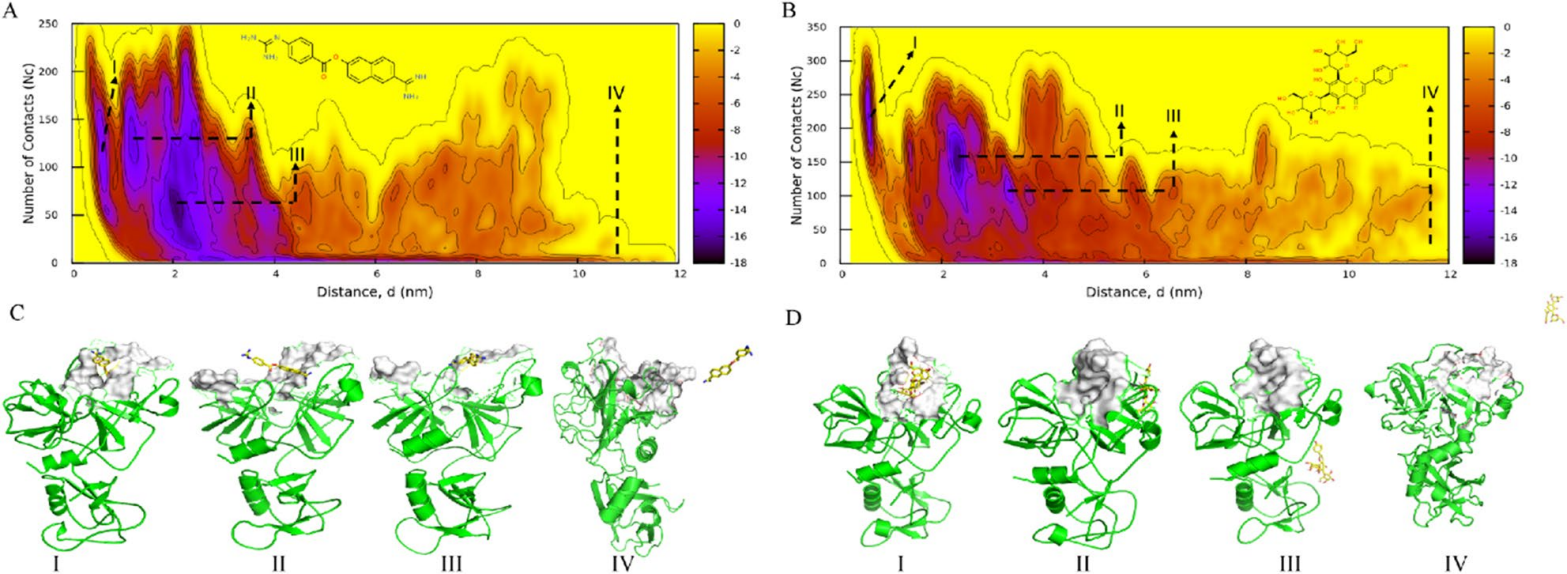
(**A**) 2D binding free energy surface of nafamostat, (**B**) 2D binding free energy surface of vicenin-2, (**C**) dissociation process of nafamostat with TMPRSS2, (**D**) dissociation process of vicenin-2 with TMPRSS2. Different states of the dissociation process of the nafamostat and vicenin-2 concerning the active site of TMPRSS2 (surface representation) along the free energy surface (depicted by the Roman letters I–IV). The contour lines depicts the binding free energy in kcal/mol.

The two-dimensional (2D) binding free energy surface contour plot and different conformational states of nafamostat and vicenin-2 during the ligand dissociation process from the active site of TMPRSS2 are shown in Fig. 7A, B respectively. The binding free energy of nafamostat obtained through well-tempered metadynamics simulations is − 16.4 kcal/mol. The average structure of the ligand dissociation process is represented from states I to IV. Nafamostat binding mode at the active site is represented by the average structures of state I (d = 0.62 nm; Nc = 125), followed by state II (d = 1.20 nm; Nc = 125) and state III (d = 2.06 nm; Nc = 50) which represent the intermediate states of the ligand dissociation process and the State IV represents the completely dissociated state (Fig. 7C).

The binding free energy of vicenin-2 is − 16.3 kcal/mol, which is very close to that of nafamostat, indicating that vicenin-2 could be a promising lead molecule for the inhibition of TMPRSS2 activity. The different states of the dissociation process of vicenin-2 are also shown in Fig. 7D. State I is the average binding mode of vicenin-2 at the active site (d = 0.56 nm; Nc = 217). State II (d = 2.31 nm; Nc = 160) and state III (d = 3.3 nm; Nc = 110) represent the intermediate states during the ligand dissociation process and State IV represents the completely dissociated state (Fig. 7D). The 2D binding free energy surfaces of naringin, rhoifolin, and neohesperidin are shown in Supplementary Figure 4, and the different states of the ligand dissociation process are also mentioned, along with the collective variable values representing each state.

Well-tempered metadynamics simulation suggests that vicenin-2 is a promising lead molecule that could be further evaluated for its inhibitory potency against SARS-CoV-2. The other leading phytocompounds, such as neohesperidin, naringin, and rhoifolin are also worth studying further to evaluate their antiviral efficacy. Our study provides *in-silico* evidence supporting the potential effectiveness of vicenin-2 targeting TMPRSS2. The proposed mechanism involves the inhibition of the TMPRSS2 activity of the receptor, which is critical for SARS-CoV-2 entry into host cells.

## Materials and methods

### Homology modelling

The FASTA sequence of TMPRSS2 (UniProt ID: O15393) was downloaded from the UniProt database (https://www.uniprot.org/) and used to model the ectodomain of TMPRSS2. The modeling was done using a similarity search and subsequent template alignment against the TMPRSS2 ectodomain sequence. The PDB ID 7MEQ exhibiting high similarity to the TMPRSS2 receptor was taken as the template and modeled by the consensus-based structure prediction method. After homology modeling, the modeled structure was subjected to structure refinement using the Prime module of Schrodinger software. The modeled structure was further validated by the Ramachandran plot and Protein Reports Module in Maestro^46–48^.

### Protein preparation and receptor grid generation

Protein preparation was carried out in the Protein Preparation Wizard^35^ of the Schrodinger Suite 11.4 (Maestro package version 11.4 from Schrödinger, LLC) with the TMPRSS2 ectodomain model. During the time of preparation, all the misaligned features were assigned, bond orders, hydrogen, disulfide bonds, and zero-order bonds to metals were added and water molecule within 5 Å in the hetero groups was removed. The H-bond network was optimised by sampling 180 flips of the terminal chi angle for Asn, Gln, and His. This substantially alters the spatial H-bonding capabilities of the side chains, but it does not significantly alter the fit to the electron density. The two His tautomers (proton on either the Nδ or Nε nitrogen) were sampled, along with the neutral and protonated states of Asp, His, and Glu. Moreover, hydrogens on thiols and hydroxyls were sampled to maximise the H-bond network. Depending on the complexity of the H-bond networks, the ProtAssign algorithm can be executed in either a "standard" mode, which typically takes a few seconds, or an "exhaustive" mode, which encompasses a greater number of states and can be executed for minutes or hours. The standard mode conducts Monte Carlo sampling for clusters with more than 100 possible states and comprehensive sampling of all states for H-bond clusters with up to 100 combinations. The exhaustive mode conducts Monte Carlo sampling for clusters with more than 10,000 possible states and sampling of all states for H-bond clusters with up to 10,000 combinations. After pre-processing, the structure refinement was performed, followed by a brief relaxation of the structure was performed with the Impact Refinement module (Impref), where all-atom-constrained minimization was done using the OPLS4 force field to relieve the steric clashes until the RMSD cut-off of 0.30 Å was reached^35^. Then a receptor grid was built around the active site residues of TMPRSS2 (Ser441-His296-Asp345) using the Glide Module of Maestro^38,39^.

### e-pharmacophore hypothesis and ligand screening

An e-pharmacophore model was developed based on the spatial arrangement of the active site residues of TMPRSS2 to identify the pharmacophore features. Ligands were selected from the natural product database LOTUS^37^, ChEMBL (https://www.ebi.ac.uk/chembl/) database, OTAVA chemicals (https://www.otavachemicals.com/), and structurally optimized before screening at the physiological pH of 7.1 using the ligprep module of the Schrodinger suite. All plausible stereoisomers and tautomers of the ligands were generated and energy minimization of the ligands was done using the OPLS4 force field. Virtual screening based on the e-pharmacophore hypothesis was conducted to identify the lead compounds with optimal structures binding to the TMPRSS2 active site using the phase module of the Schrodinger suite. The fitness scores of the compounds were used to select the best hits from the e-pharmacophore screening^48,49^.

### Virtual screening and molecular dynamics simulations

The virtual screening was carried out using the Phase module of the Schrodinger Suite. The best docking pose obtained from the XP docking was used for the MD simulations. Selected hits from the e-pharmacophore-based screening and docking were subjected to 300 ns MD simulations using the Desmond module of Schrodinger, the TIP3P water model^50^ and the OPLS4 force field. An orthorhombic TIP3P water box shielded the whole protein–ligand complex, and the solvated system was neutralized by adding Na^+^/Cl^-^. Using Nose–Hoover temperature^51,52^ coupling and isotropic scaling, the temperature and the pressure of the system were maintained at 300 K and 1 atm, respectively. The trajectory of intermediate structures was saved at every 100 ps and used for further analyses^53^. By using the simulation interaction diagram wizard in the Desmond module protein-ligand interaction; RMSD and RMSF plots were generated.

### RMSD and RMSF analysis

Root Mean Square Deviation (RMSD) is a statistical tool for calculating the average change in displacement of a set of atoms for a given frame relative to the reference frame. It has been calculated for every frame in the trajectory. For frame x, the RMSD is

$${\text{RMSD}}_{x} = \sqrt {1/N\mathop \sum \limits_{i = 1}^{N} \left( {r_{i}^{\prime} \left( {t_{x} } \right) - r_{i} \left( {t_{ref} } \right)} \right)^{2} }$$

where t_ref_ is the reference time (usually the first frame is used as the reference and is treated as time t = 0); N is the number of atoms in the atom selection; where r'_i_ is the position of the chosen atoms in frame x following superimposition on the reference frame, where frame x is recorded at time t_x_. For every frame in the simulation trajectory, the process is repeated.

Similarly, Root Mean Square fluctuation (RMSF) is used for characterizing the local changes along the protein chain. For residue i, the RMSF is:

$${\text{RMSF}}_{{\text{i}}} = \sqrt {1/T\mathop \sum \limits_{t = 1}^{T} \left\langle( {r_{i}^{\prime} \left( t \right) - r_{i} \left( {t_{ref} } \right)} \right) \rangle^{2} }$$

where r_i_ is the position of residue i; r'_i_ is the position of atoms in residue i after superposition on the reference, t_ref_ is the reference time; T is the trajectory time over which the RMSF is calculated; and the angle brackets denote that the average of the square distance is taken over the selection of atoms in the residue (Schrodinger, LLC, New York, NY, USA).

### Binding free energy analysis

The free energy of binding was predicted through the prime MM-GBSA^54^ module of the Maestro program using the OPLS4 force field^36,55^. Protein residues beyond 12 Å from the ligand-bound area were frozen during energy minimization, and the binding energy was calculated using the equation given below. To determine the binding free energy of receptor-ligand binding (ΔG_bind_), subtract the free energies of the unbound receptor (G_protein_) and ligand (G_ligand_) from the receptor-ligand complex (G_complex_).

1 $$\Delta {\text{G}}_{{{\text{bind}}}} = {\text{ G}}_{{{\text{complex}}}} - \, \left( {{\text{G}}_{{{\text{protein}}}} + {\text{ G}}_{{{\text{ligand}}}} } \right)$$

The ΔG_bind_ is composed of the changes in the molecular mechanical gas phase energy (ΔEMM), entropy contribution, and solvation-free energy.

where G_complex_ is the whole free energy of the protein–ligand system, and G_protein_, G_ligand_ is the gross free energies of the protein and ligand. The value of G_protein_, G_ligand_ is evaluated by

2 $${\text{Gx}} = {\text{EMM}} - \left( {{\text{TS}}} \right) + \left( {{\text{G}}_{{{\text{solvation}}}} } \right)$$

where EMM is the average molecular mechanics potential energy in the vacuum. (TS) denotes the product of the entropic contribution and temperature, and (G_solvation_) is the free energy of solvation. Further,

3 $${\text{EMM}} = {\text{E}}_{{{\text{bonded}}}} + {\text{E}}_{{{\text{non}} - {\text{bonded}}}} = {\text{E}}_{{{\text{bonded}}}} + \left( {{\text{E}}_{{{\text{vdW}}}} + {\text{E}}_{{{\text{elect}}}} } \right)$$

where E_bonded_ is bonded interactions, and E_non-bonded_—includes van der Waals’ (E_vdW_) and electrostatic (E_elect_) interactions and are modelled using a Lennard–Jones (LJ) and Coulomb potential function, respectively.

G_solvation_ (solvation free energy) is given by:

4 $${\text{G}}_{{{\text{solvation}}}} = {\text{G}}_{{{\text{polar}}}} + {\text{G}}_{{{\text{non}} - {\text{polar}}}}$$

here, G_non- polar_ and G_polar_ are the non-electrostatic and electrostatic contributions to the solvation-free energy, respectively. G_non- polar_ includes attractive and repulsive forces between solute and solvent that are generated by van der Waals’ interactions and cavity formation, respectively. Furthermore,

5 $${\text{G}}_{{{\text{non}} - {\text{polar}}}} = {\text{G}}_{{{\text{cavity}}}} + {\text{G}}_{{{\text{vdW}}}}$$

MM-GBSA calculates the total free energy of interaction between ligand and receptor molecule by combining the nonpolar solvation term (GNP), molecular mechanics energies (EMM), and surface generalized born solvation model for polar solvation (GSGB).

6 $${\text{G }} = {\text{ EMM }} + {\text{ GSGB }} + {\text{ GNP}}$$

### Principal component analysis

PCA is a multivariate statistical technique applied to reduce the number of dimensions needed to describe protein dynamics through a decomposition process***.*** PCA is frequently used to reduce the dimensionality of MD data and identify predominant modes of protein motion. An eigenvalue decomposition (EVD) of the covariance matrix leads to a complete set of orthogonal collective modes (eigenvectors), each with a corresponding eigenvalue (variance) that characterizes a portion of the motion. The covariance matrix is typically a 3 m × 3 m real, symmetric matrix, where m is the number of residues. EVD results in 3 m eigenvectors (modes) and 3 m—6 non-zero corresponding eigenvalues, provided at least 3 m observations are used^41^. When plotted against the mode index, a "scree plot" typically appears, allowing for a large portion of protein motions to be captured with a small number of modes defining a low-dimensional subspace. For proteins, 20 modes are usually enough to define an "essential space" that captures the motions governing biological function, achieving a significant reduction in dimension.

### Dynamics cross-correlation analysis

The Dynamic Cross-Correlation Matrix (DCCM) is used in MD simulations to study collective movements and correlations among atoms or atom groups within a biomolecular system^56^. It helps to understand the dynamic behavior and communication between different regions of the protein. The matrix element C_ij_ in DCCM reads as:

$${\text{C}}_{{{\text{ij}}}} = \left\langle {\Delta \overrightarrow {{r_{i} }} \left( t \right)\Delta \overrightarrow {{r_{j} }} \left( t \right)} \right\rangle /{\left(\left\langle{\Delta \overrightarrow {{r_{i} }} \left( t\right) { }} \right\rangle^{2} \left\langle{\ \Delta \overrightarrow {{r_{j} }} \left( t \right) { }} \right\rangle^{2}\right)}^{1/2}$$

where <> represents an MD-averaged quantity and $\Delta \overrightarrow {{r_{i} }} \left( t \right) = \left\langle {\overrightarrow {{r_{i} }} \left( t \right)} \right\rangle - \overrightarrow {{r_{i} }} \left( t \right)$ the displacement from its average MD position $\overrightarrow {{r_{i} }} \left( t \right)$ of atom i during a generic MD step. C_*ij*_ values range from − 1.0 for entirely anticorrelated movements to + 1 for completely correlated motions. A number close to + 1 indicates a strong link between the movements of two C_α_ atoms. The highest values are seen for C_α_ atoms in residues i and i ± a, where a = 0, 1, 2 (diagonal elements in the map)^57^.

### Protein–protein docking

The spike protein (PDB ID: 6CS2) and the TMPRSS2 model were docked using the protein–protein docking web server ClusPro 2.0^58^. The cleavage site of the viral spike protein was defined as active residues for docking protocol. The substrate binding site and the catalytic triad were defined as the active residues for TMPRSS2. Further, molecular docking with high-scored phytocompounds identified through virtual screening was used to dock with TMPRSS2-Spike protein complex using Schrodinger’s GLIDE module^38,39^.

### Validation of molecules using well-tempered metadynamics simulation

Well-tempered metadynamics simulations were further used to validate the selected molecules obtained after docking and all-atom MD simulations^59^. CHARMM General Force Field (CgenFF)^60^ was used for all small molecules and the CHARMM36 all-atom force field was used for the proteins with the CHARMM-modified TIP3P water model^50^. The protein–ligand complex obtained after 300 ns all-atom MD simulations was solvated using TIP3P water molecules in a cubic box with dimensions of 9.4 × 9.4 × 9.4 nm^3^_,_ neutralized, and maintained at 150 mM NaCl. Initial energy minimization was performed using the steepest descent algorithm^61^ and followed by equilibration at 300 K using Berendsen thermostat^62^ for 5 ns in a constant number of particles, volume, and temperature (NVT) ensemble, another 5 ns at a constant number of particles, temperature, and pressure (NPT) ensemble using Berendsen thermostat and Parrinello-Rahman barostat^63^. During the NVT and NPT equilibrations, all the heavy atoms were restrained with a force constant of 25 kcal/mol. Also, an unrestrained equilibration was performed for 5 ns in the NPT ensemble. Particle Mesh Ewald (PME)^64^ was used for electrostatics with a distance cut-off of 12 Å. The simulations were conducted with a 2 fs time step, and the equilibrated TMPRSS2-ligand complexes were subjected to well-tempered metadynamics simulations for free energy calculations using GROMACS 2023.2 software coupled with the PLUMED 2-2.9.0 plugin^59,65,66^.

### Well-tempered metadynamics simulation

Well-tempered metadynamics simulations were performed to calculate the binding free energies of the selected ligands bound to TMPRSS2. The simulations used two collective variables: distance (d) and the number of contacts (Nc). The collective variable distance (d) refers to the distance between the COM (Centre of Mass) of the amino acids in the active site of TMPRSS2 and the COM of the ligand. The distance was calculated using residues within 8 Å of the ligand's active site (residues 294–300, 383–398, 427–443, and 459–471). The number of contacts were calculated within 4 Å surrounding the ligand’s atoms. The hill height was optimized as 0.2 kJ/mol with a Gaussian width of 0.06 Å for D and 5.0 for Nc with a bias factor of 15 and a hill deposition rate of 2 ps. The binding free energy was calculated from the HILLS files generated during the simulations using the sum_hils program in PLUMED software. The 2D free energy surface was plotted using the gnuplot. Cluster analysis was performed at the centre of each of the free energy wells on the free energy surface based on the respective collective variables and average structures of those clusters representing the minimum energy conformation were extracted for protein–ligand interaction analysis.

## Conclusion

The SARS-CoV-2 virus poses a significant global threat to economies and public health due to its rapid spread and highly contagious nature. The SARS-CoV-2 infection was initiated by the interaction of the host TMPRSS2 and the viral spike protein, presenting an opportunity for the therapeutic development of viral entry inhibitors. Targeting host proteases in viral infections is a novel strategy for developing antiviral drugs that may overcome resistance. TMPRSS2, a host enzyme essential for proteolytic activation and pathogenesis of coronaviruses, is a promising drug target for treating viral infections. Small-molecule therapies can stop viral replication following infection, and our study proposes that vicenin-2, neohesperidin, naringin, and rhoifolin can act as TMPRSS2 antagonists and viral entry inhibitors, subject to experimental validations. Vicenin-2, neohesperidin, naringin, and rhoifolin have a better binding affinity towards the TMPRSS2 active site than hesperidin, which displayed larger RMSD values during the MD simulations. An efficient, computationally driven drug repurposing strategy is presented here for identifying potential TMPRSS2 inhibitors, with docking and MD simulations providing structural insights into their mechanism of binding and the well-tempered metadynamics further confirmed the interaction of these ligands between the TMPRSS2 and viral spike protein.

### Supplementary Information

Supplementary Information.

## Data Availability

The datasets used and/or analysed during the current study available from the corresponding author on reasonable request.
