# Supplementary material for: Computational screening combined with well-tempered metadynamics simulations identifies potential TMPRSS2 inhibitors
Source: Sci Rep. 2024 Jul 13;14:16197. doi: 10.1038/s41598-024-65296-7 (PMC11246518; doi:10.1038/s41598-024-65296-7)
Supplement: Supplementary file 1 — Supplementary Information. [file 41598_2024_65296_MOESM1_ESM.docx]

**Computational screening combined with well-tempered metadynamics simulations identifies potential TMPRSS2 inhibitors**

Sharanya CS^a^, Wilbee DS^a,b^, Shijulal Nelson Sathi^c^ and Kathiresan Natarajan^a⃰^

^a^ Transdisciplinary Biology, Rajiv Gandhi Centre for Biotechnology, Thiruvananthapuram, Kerala, India

^b^ College of Pharmaceutical Sciences, Govt Medical College, Thiruvananthapuram, Kerala, India

^c^ Bioinformatics Laboratory, Rajiv Gandhi Centre for Biotechnology, Thiruvananthapuram, Kerala, India

⃰ Corresponding author: E-mail: kathiresan@rgcb.res.in


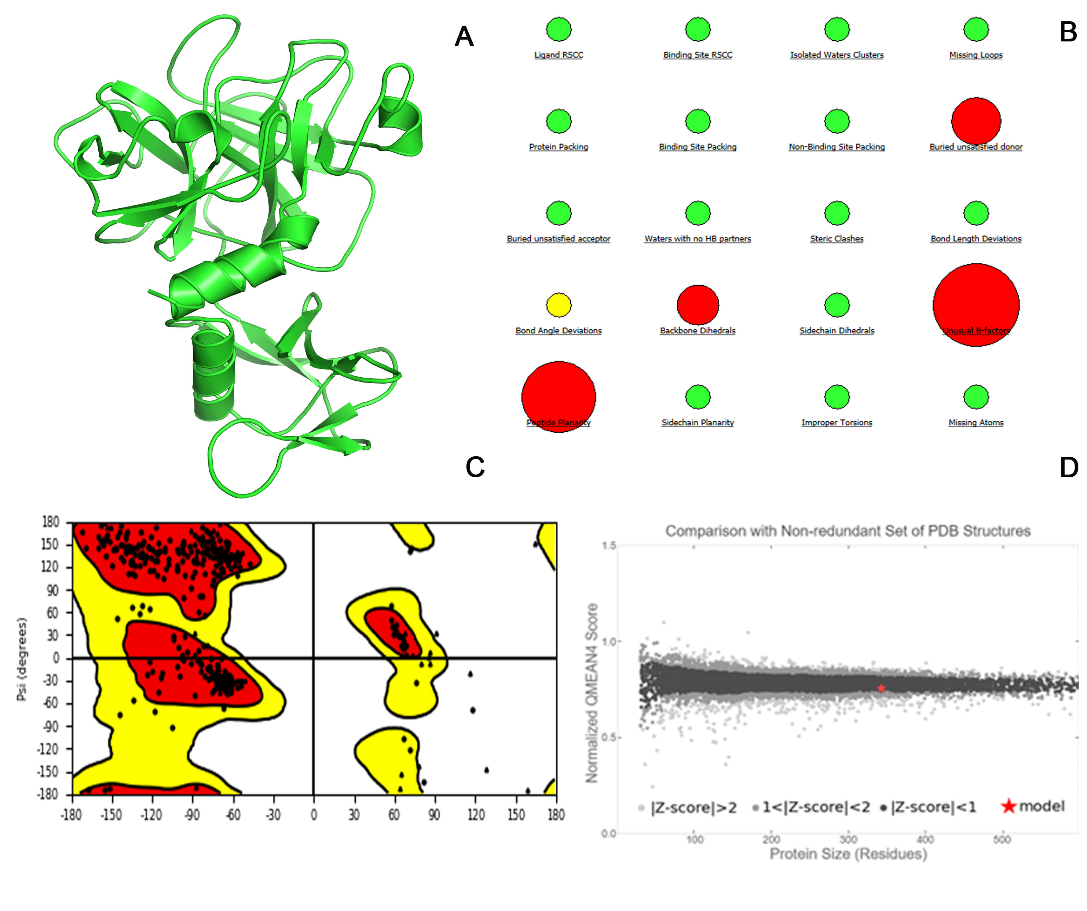

**Supplementary Figure 1:** Predicted structure of TMPRSS2. (A) Built model of TMPRSS2 monomeric protein using PDB ID: 7MEQ as the model template (B) Protein reliability report (C) Ramachandran plot showing the distribution of 95.03% TMPRSS2 residues in the most favoured region. (D) Normalised Qmean4 score compared to the non-redundant set of PDB structures


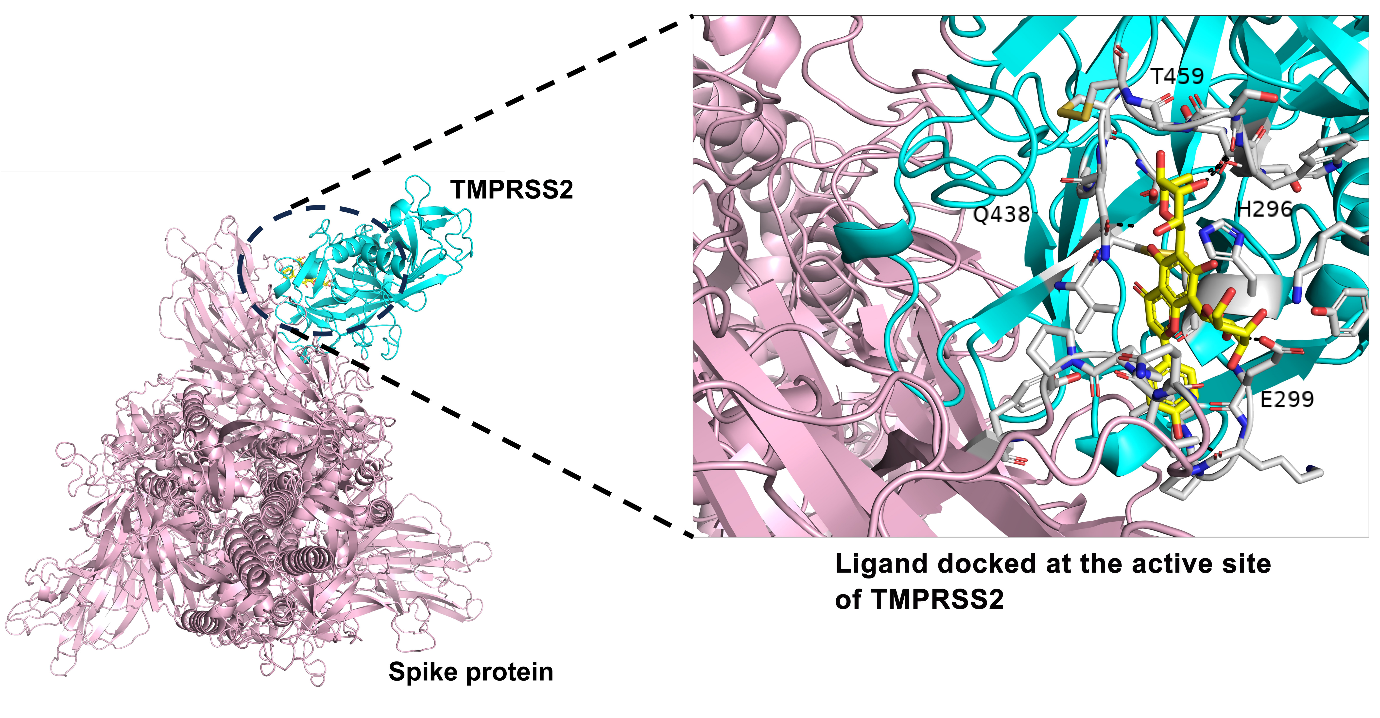


**Supplementary Figure 2:** Structure of spike protein-TMPRSS2 complex docked with vicenin-2.





**Supplementary Figure 3:** One-dimensional free energy profile of TMPRSS2-ligand complexes.


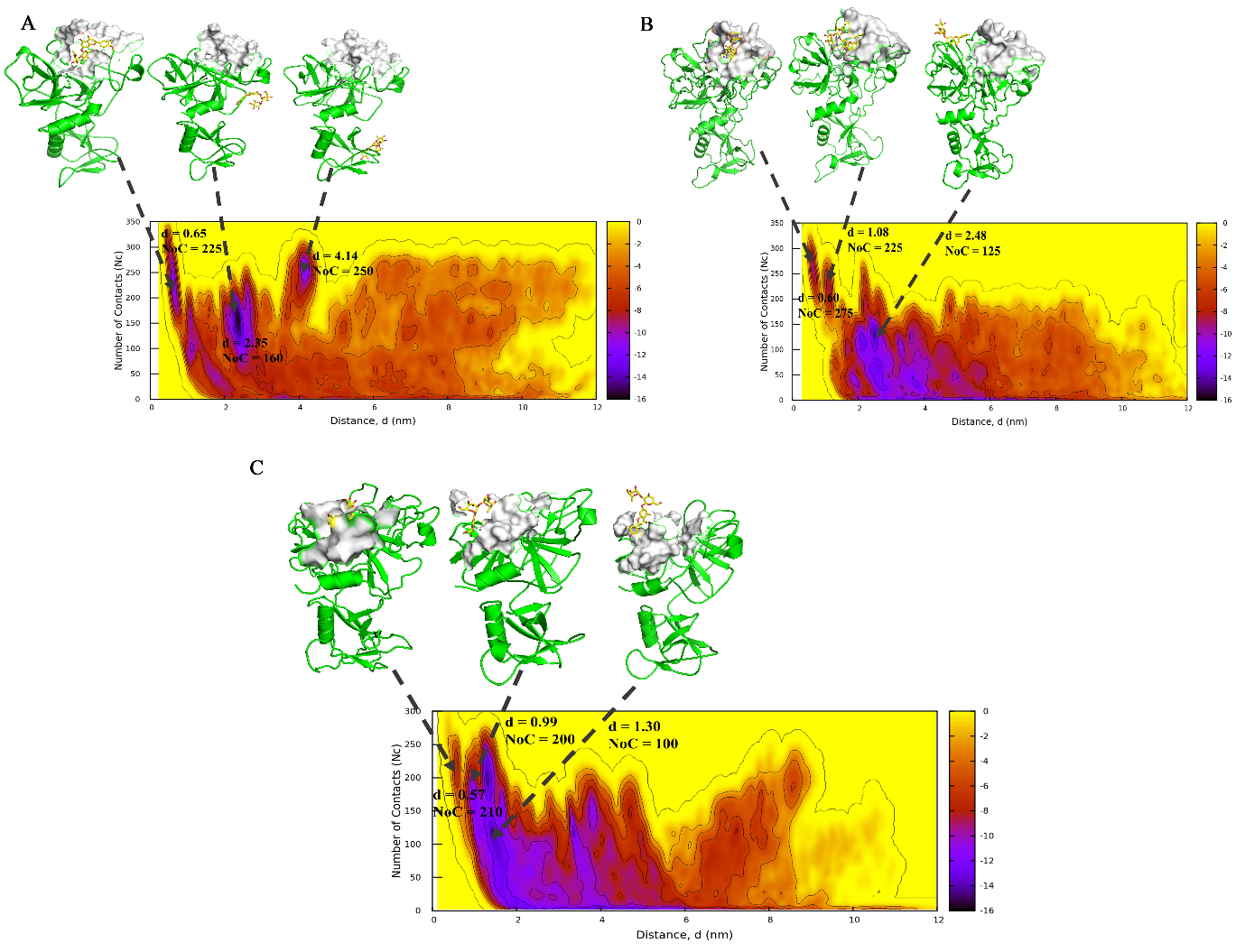


**Supplementary Figure 4:** 2D binding free energy surface and different states of (A) neohesperidin, (B) naringin and (C) rhoifolin during the dissociation process from the active site of TMPRSS2 (surface representation) along the free energy surface with respect to collective variables Distance, d and number of contacts, Nc.
